# A Theory-Informed, Personalized mHealth Intervention for Adolescents (Mobile App for Physical Activity): Development and Pilot Study

**DOI:** 10.2196/35118

**Published:** 2022-06-10

**Authors:** Alex Domin, Arif Uslu, André Schulz, Yacine Ouzzahra, Claus Vögele

**Affiliations:** 1 Research Group: Self-Regulation and Health Department of Behavioural and Cognitive Sciences University of Luxembourg Esch-sur-Alzette Luxembourg; 2 Research Support Department University of Luxembourg Esch-sur-Alzette Luxembourg

**Keywords:** mobile health, physical activity, app, adolescents, within-subject, mHealth, sedentary behavior, behavior change techniques, BCTs, Fitbit, mobile phone

## Abstract

**Background:**

Evidence suggests that physical activity (PA) during childhood and adolescence is crucial as it usually results in adequate PA levels in adulthood. Given the ubiquitous use of smartphones by adolescents, these devices may offer feasible means to reach young populations and deliver interventions aiming to increase PA participation and decrease sedentary time. To date, very few studies have reported smartphone-based interventions promoting PA for adolescents. In addition, most available fitness apps do not include the latest evidence-based content.

**Objective:**

This paper described the systematic development of a behavior change, theory-informed Mobile App for Physical Activity intervention with personalized prompts for adolescents aged 16 to 18 years. The within-subject trial results provided the first evidence of the general effectiveness of the intervention based on the outcomes step count, sedentary time, and moderate to vigorous PA (MVPA) minutes. The effectiveness of the intervention component *personalized PA prompt* was also assessed.

**Methods:**

A 4-week within-subject trial with 18 healthy adolescents aged 16 to 18 years was conducted (mean age 16.33, SD 0.57 years). After the baseline week, the participants used the Mobile App for Physical Activity intervention (Fitbit fitness tracker+app), which included a daily personalized PA prompt delivered via a pop-up notification. A paired 1-tailed *t* test was performed to assess the effectiveness of the intervention. Change-point analysis was performed to assess the effectiveness of a personalized PA prompt 30 and 60 minutes after prompt delivery.

**Results:**

The results showed that the intervention significantly reduced sedentary time in adolescents during the first week of the trial (*t*_17_=−1.79; *P*=.04; bootstrapped *P*=.02). This trend, although remaining positive, diminished over time. Our findings indicate that the intervention had no effect on metabolic equivalent of task–based MVPA minutes, although the descriptive increase may give reason for further investigation. Although the results suggested no overall change in heart rate–based MVPA minutes, the results from the change-point analyses suggest that the personalized PA prompts significantly increased heart rate per minute during the second week of the study (*t*_16_=1.84; *P*=.04; bootstrapped *P*=.04). There were no significant increases in participants’ overall step count; however, the personalized PA prompts resulted in a marginally significant increase in step counts per minute in the second week of the study (*t*_17_=1.35; *P*=.09; bootstrapped *P*=.05).

**Conclusions:**

The results of the trial provide preliminary evidence of the benefit of the Mobile App for Physical Activity intervention for modest yet significant reductions in participants’ sedentary time and the beneficial role of personalized PA prompts. These results also provide further evidence of the benefits and relative efficacy of personalized activity suggestions for inclusion in smartphone-based PA interventions. This study provides an example of how to guide the development of smartphone-based mobile health PA interventions for adolescents.

## Introduction

### Background

The beneficial impact of physical activity (PA) has been extensively documented, showing improved physical and mental health across the life span together with increased life expectancy [1]. In contrast, lack of PA and increased sedentary time continue to represent a serious public health burden. Low PA levels are associated with a higher prevalence of chronic diseases (eg, coronary heart disease, type 2 diabetes, breast cancer, and colon cancer) and an increased risk of morbidity and mortality, accounting for >5.3 million premature deaths annually [2]. In European Union countries, alarming levels of physical inactivity have been observed in adolescents [1]. As indicated by the Organisation for Economic Co-operation and Development report *Health at a Glance: Europe 2020*, less than 25% of boys and 20% of girls show sufficient levels of self-reported PA at the age of 15 years [3].

There is evidence that PA during childhood and adolescence is crucial as it usually results in adequate PA levels in adulthood [1]. Therefore, it is an essential research and public health priority to increase PA participation and decrease sedentary time in adolescents. Specific attention should be devoted to adolescents aged 16 to 18 years as they show the lowest absolute PA levels among children and young people aged 5 to 19 years [4]. Recent advances in digital technology have the potential to be successfully used in interventions aiming to improve PA-related outcomes in adolescents. In this study, we used the rationale that mobile devices have the potential to help their users engage in and adhere to different types of PA in several contexts (eg, school, leisure time, and transportation). In addition, mobile health (mHealth) interventions have the potential to adaptively respond to individuals’ actions and states and deliver intervention options *just in time* (ie, when and where they are most appropriate [5,6]). Such just-in-time adaptive interventions (JITAIs) can facilitate health behavior change at times of both need for behavior support and receptivity [7]. Given the ubiquitous use of smartphones by adolescents, these devices may offer feasible means to reach young populations and deliver interventions aiming to increase PA participation and decrease sedentary time. Previous research suggests that an automated advice system may be as productive as or even preferable to a human advisor for increasing PA participation [8,9]. Preliminary evidence suggests that cardiorespiratory fitness gains can be sustained using a dedicated smartphone app [10-12]. The literature on JITAIs in particular shows mixed yet promising evidence. Specifically, a review found mixed evidence for JITAI effects on behavior, but no study was sufficiently powered to detect any effects. Another study reported that the JITAI condition demonstrated a significant improvement in health over the wait-list control condition [13,14]. In addition to interventions based on smartphone apps potentially being efficacious, they may also come with reduced costs in comparison with interventions involving face-to-face interviewing and guidance.

Although numerous fitness apps for smartphones address PA participation and sedentary behavior, most of them do not include the latest evidence-based content [15]. A recent review concluded that, despite not being grounded in theory, some interventions contain one or more behavior change components or behavior change techniques (BCTs) [16]. However, the systematic implementation of BCTs in dedicated apps has rarely been achieved. Michie et al [17] argue that many interventions applying recommended BCTs are not designed systematically and are theory-inspired rather than theory-based. This may result in low participant engagement with the intervention and a lack of longitudinal effects. Therefore, it is crucial to implement BCTs systematically while evaluating not only the effectiveness of the complete intervention but also the effectiveness of its smallest components. In a recent scoping review, we argued that the efficacy of smartphone-based mHealth PA interventions can be considerably improved through a more systematic approach of developing, reporting, and coding the interventions [18]. Therefore, in this study, we try to build on the previous theoretical findings systematically to maximize the impact of our intervention.

Smartphone-based behavior change interventions are typically tested using randomized controlled trials (RCTs) [19]. Although RCTs provide the highest level of scientific evidence, they evaluate the effect of an intervention as a whole. Therefore, RCTs would typically not provide information regarding which components work best and what factors modulate their efficacy. Considering the critical role of factors such as the timing of administration and the context in which components are implemented [6], an RCT may not provide the level of detail required to appropriately assess the efficacy of smartphone-based interventions. Finally, RCTs are quite long, averaging 5.5 years from recruitment to publication date [20,21]. Therefore, it was suggested to implement alternative designs that may provide prompter and more relevant answers while being more suitable to the research question. Trial designs where participants serve as their own controls, also known as within-subject designs, can reduce the number of study participants needed to detect outcomes and accelerate the research process while also simplifying the study procedures [22]. In addition, such designs have a much shorter duration and allow for the testing of the efficacy of individual components [6]. Therefore, an mHealth PA intervention for adolescents may benefit from the implementation of such an evaluation design.

Personalization of intervention components has been shown to be important for their overall success as it may significantly affect the to date unresolved challenge of mHealth intervention engagement of the participants and, as a result, the outcomes of the intervention [23]. However, most current mHealth apps do not often offer personalized features although they could be important in increasing motivation and engagement. Examples of personalization include but are not limited to differentiation between habitual and unforeseen behaviors, collection of information about preferred PA, and activity suggestions depending on the current location or daily schedule [23,24]. Therefore, to maximize the potential of the intervention, it is important that the intervention components are personalized for its participants.

Previous studies mainly centered on the adult population have demonstrated that tackling the aforementioned gaps might be beneficial. Bond et al [25] developed and tested a smartphone-based intervention with a dedicated smartphone app, which significantly reduced sedentary behavior time over 4 weeks. Rabbi et al [23] and Klasnja et al [24] designed smartphone-based interventions that demonstrated preliminary evidence of the efficacy of personalized PA suggestions that are contextualized to the user’s previous behavior and environment. Kramer et al [26] conducted a microrandomized trial (MRT) reporting a significant step goal increase triggered by cash incentive components. Finally, Gaudet et al [27] used a minimalist PA Fitbit tracker–based intervention with adolescents aged 13 to 14 years, which resulted in increased PA.

### Objectives

This project aimed to further the findings from previously conducted studies while concentrating on the adolescent population. The Mobile App for Physical Activity intervention uses a personalization feature through setting individualized PA goals and delivering tailored feedback based on the individual’s performance not limited solely to step count. The Mobile App for Physical Activity intervention was developed based on the Behaviour Change Wheel framework [28] by applying an approach that incorporates findings from qualitative studies and recommended and efficacious BCTs based on systematic reviews and meta-analyses. Finally, the research available to date tends to focus on the development of apps for adults rather than adolescents. Although adolescents aged 16 to 18 years represent a highly relevant target group for improving PA participation, only very few studies have addressed this particular age group [18]. To the best of our knowledge, this is the first study to develop a behavior change PA mHealth intervention with personalized prompts for adolescents aged 16 to 18 years evaluated using a within-subject experimental design.

The Mobile App for Physical Activity within-subject trial was conducted with the objective to provide the first evidence of the general effectiveness of the Mobile App for Physical Activity intervention among adolescents based on the outcomes of step count, sedentary time, and moderate to vigorous PA (MVPA) minutes. The effectiveness of the intervention component *personalized PA prompt* was also assessed using change-point analyses to determine whether similar PA smartphone-based interventions could benefit from the implementation of such a component.

We hypothesized that (1) the Mobile App for Physical Activity intervention would decrease the daily sedentary time of adolescents during the intervention in weeks 1, 2, and 3 compared with baseline measurements (primary variable of interest). We also hypothesized that (2) there would be an increase in the time spent in MVPA minutes and the number of daily steps in weeks 1, 2, and 3 compared with baseline measurements (secondary variables of interest). Finally, we hypothesized that (3) the participants’ step count and heart rate (HR) would show an increase after the delivery of a personalized PA prompt.

## Methods

### The Mobile App for Physical Activity Intervention

Mobile App for Physical Activity is an intervention developed to promote PA among adolescents aged 16 to 18 years. It was aimed at adolescents who showed insufficient PA levels according to the World Health Organization (WHO) recommendations (ie, <60 minutes of moderate or vigorous PA each day, associated with 11,700 steps daily [29]) but were interested in increasing it. This minimalist, multicomponent mHealth PA intervention combines a Fitbit smartphone app [30], personalized assistance, and a wrist-worn activity tracker (Fitbit Charge 4) [31] that collects HR-based Active Zone Minutes or MVPA minutes, active minutes based on the metabolic equivalent of task (MET) or MVPA minutes, step count, and sedentary minutes based on MET data. The Mobile App for Physical Activity intervention includes basic features of the Fitbit app and tracker as well as additional personalized assistance features—daily personalized PA prompts, weekly goal adjustment, and interactive assistance realized via the chat messaging feature—to help participants resolve any problems concerning achieving daily goals or using the intervention components. All additional components were implemented just in time by AD using the back-end features of the intervention (the Fitabase platform and Fitbit web interface). In total, 4 different outcome measures were controlled for following the recommendations of Thompson et al [32] suggesting the consideration of a multidimensional PA user profile. This device was selected for several reasons. We were interested in a device that would be, on the one hand, attractive yet unnoticeable and the least burdensome (to support user engagement) yet, by contrast, able to collect HR data (for MVPA minute calculation based on HR data in addition to MET values) and that would track and automatically recognize various types of PA performed by users. The triaxial accelerometer produced by ActiGraph is considered the gold standard in PA measurement, currently proposing several wrist-worn activity trackers [33]. However, the proposed HR measurement method would have necessitated additional wireless devices, which may be considered burdensome by users. In addition, activity recognition was not automated. The latest reviews have reported satisfactory validity, reliability, and feasibility of consumer-grade activity trackers produced by Fitbit and other companies [34,35]. After analyzing the market of commercial activity trackers, we identified the device that matched most of our requirements—Fitbit Charge 4, a small, wrist-worn, waterproof activity tracker with an inbuilt photoplethysmographic sensor to assess HR and automatic recognition of 7 types of PA. Similar Fitbit devices have already been used in mHealth PA interventions for data collection [27,36-40]. The limitation that we encountered with this device related to data received from the Fitbit server—MVPA minutes or, according to Fitbit, *activity minutes* (calculated based on MET values) and *Active Zone Minutes* (calculated based on HR values) were provided *as is* (ie, without providing an algorithm based on which MVPA minutes were calculated). To date, the literature presents mixed evidence on the validity of HR (–3% to +3% error rates), maximal aerobic capacity (VO_2max_), and energy expenditure measurements [41] yet confirms the relative validity of the Fitbit MVPA minute calculation [42], accurate recognition of the PA type [43], excellent interinstrument reliability, and good levels of agreement between devices [44]. On the basis of these considerations, we decided to use the Fitbit Charge 4 as a primary data collection device taking into account the limitations described.

The Fitbit app presents the user with a large set of features and tools that can be displayed in a selective manner. To use this software as a part of the Mobile App for Physical Activity intervention, the functionality of the Fitbit app was used as a toolkit and was tweaked to only make use of the features selected below. Therefore, the next constituent of the Mobile App for Physical Activity intervention included a combination of the Fitbit smartphone app and personalized assistance, which included both *push* and *pull* intervention components.

The first pull component was *graphs and stats*. This component, delivered via the home page of the Fitbit app, presents users with graphical feedback on their daily goals with the potential for personalization. Specifically, users can compare the results attained thus far with their personalized goals. Each participant was assigned 2 personalized daily goals: a step count goal and an *Active Zone Minutes* goal. These goals were set individually via remote Fitbit account access at the end of every week based on a 5% increase from the average daily step or Active Zone Minute count achieved during the previous week. A 5% increase mark was chosen to provide a substantial yet feasible increase goal based on the findings from the study by Degroote et al [45]. If a participant underperformed, the daily step or Active Zone Minute goals remained the same.

The second pull component was *interactive assistance*. Through a dedicated message tab, the participants received advice or could communicate with AD in case they encountered any problems in either achieving their daily goals or using the intervention components. Web-based support was initially intended as an automated advisor providing personalized support and problem-solving strategies and answering inquiries based on a strategy grounded in artificial intelligence research. However, because of the prolonged development period, for this first version of the Mobile App for Physical Activity intervention, AD substituted an automated advice system. There is recent evidence that, while providing solutions for identified obstacles, the implementation of an automated advisor is considered promising in increasing PA even compared with human advisors. Therefore, this component should be further developed and tested in future trials [9,10,46,47]. This component also has the potential to reinforce coping planning (by providing coping responses for dealing with potential barriers and difficult situations) [26,48].

The first push component was a *personalized PA prompt*. According to the results of the 2 most recent MRTs, tailored push suggestions in a PA context were associated with greater engagement with an mHealth app and increased PA participation [24,49]. In the Mobile App for Physical Activity, the users received 1 tailored suggestion (delivered as a pop-up notification). Every day after school classes had finished (5 PM-7 PM), the participants received a personalized PA prompt via a Fitbit pop-up notification. Each message was prepared by AD considering the daily step count performance so far and its percentage correlation with the daily goal. Depending on the participant’s achievements, the message could be framed in 5 different ways according to the percentage reached compared with the personalized goal: <40%, ≥40%, ≥60%, ≥80%, and ≥100%. The message also included different PA health benefits that the participants could potentially achieve by following their activity goals based on WHO recommendations [50]. Additional attention was paid to the positive framing of the PA suggestions [47]. The Mobile App for Physical Activity intervention included >25 PA suggestion templates developed by the research team using data collected during focus group discussions. All generated suggestion templates were reviewed and edited, and additional suggestions were created to provide a sufficient number of prompts for each condition, including the personalization to such events as an exam period. An example of such a suggestion would be the following: *Hi, Bob! You did around xxxxx steps so far and reached 80% of your goal for today—good job, can you do even better? Interesting fact: physical activity benefits improved concentration, so if you are active, you may get better study results! Keep up the effort!* Therefore, depending on the participants and their performance level as well as their daily goal, messages could differ in relation to five variables: (1) participant name, (2) number of steps so far, (3) step count percentage correlation with the daily goal, (4) PA health benefits based on WHO recommendations, and (5) general message framing. This resulted in a personalized message that could potentially be followed by interactive assistance, which is a qualitatively different combination compared with the generic messages currently provided by most commercial apps. In further iterations of the Mobile App for Physical Activity intervention, this process is planned to be more automated and personalized considering contextual factors.

The second push component was *reminders to move*. Every day after classes (4 PM-9 PM), if the participants’ behavior was identified as sedentary (no steps or any other HR-increasing activity were performed), they would receive a pop-up reminder motivating them to take ≥250 steps by the end of each hour.

These approaches were designed to support the users’ self-regulation, which is recognized as a principal factor in health behavior change [51,52]. The self-monitoring and feedback strategies implemented in this intervention component are characterized as “especially helpful” and recommended for inclusion in PA promotion interventions [47].

The last intervention component was *rewards*. This component was introduced to reinforce the self-monitoring strategies used in the Mobile App for Physical Activity intervention. The strategy of providing users with rewards was recently reported to be especially helpful in increasing PA [47]. In the preliminary-held focus group discussions, adolescents identified the sports-related rewards as the most attractive. At intake, the participants were informed that rewards could be obtained based on the number of consecutively accumulated days in line with or above the personalized goals. The rewards consisted of a digital gift voucher from a local sporting goods store. The value of the voucher depended on the number of consecutively accumulated days in line with or above the personalized goals (ie, 3, 5, or 7 days of the week resulted in a €15 [US $15.97], €30 [US $31.94], or €40 [US $42.58] voucher reward, respectively). Rewards or gift vouchers were time-contingent (ie, they were delivered via email directly at the end of the week). The reward scenario aimed to reinforce users’ self-monitoring and the consequent regular performance of sufficient PA. As the Mobile App for Physical Activity intervention was intended to be available to users over a longer period (ie, after the completion of the study), incentives were projected to be effective at early stages and further gradually substituted by habitually formed self-monitoring strategies [53]. Finally, the participants were allowed to keep the activity trackers after the end of the study.

### Selection of the Intervention Components

The selection of the Mobile App for Physical Activity intervention components was guided by 2 combined approaches. The first approach included an analysis of qualitative studies (survey, interview, and focus group discussion as methods of data collection) that explored user preferences in terms of the technological functionality of PA promotion apps [54-61]. We also conducted a focus group discussion to explore the app feature preferences of adolescents. After identification of potentially advantageous and attractive features for the PA promotion app, we aligned the results with our second approach (ie, the identification and implementation of recommended, effective, and efficacious BCTs).

To identify and select such BCTs, we used the approach described by Lyons et al [62], compiling recommended BCTs from several sources in 1 list [62]. Our selection was based on the following steps: (1) successful BCTs for increasing PA in adolescents based on the meta-analysis by Brannon and Cushing for adolescents [63], (2) BCTs that predicted PA as reported in meta-analyses on PA interventions for adults [51,63-67], (3) recommendations from the systematic review by Sullivan et al [47], and (4) BCTs identified by applying the Behaviour Change Wheel framework [28,68]. We used the Acceptability, Practicability, Effectiveness, Affordability, Side-effects, and Equity criteria (worksheet 7 of the Behaviour Change Wheel manual) to identify the appropriate BCTs based on the Mobile App for Physical Activity intervention functions. As an example, the BCT *Credible source* was identified as appropriate and applied within 2 intervention components: interactive assistance and personalized PA prompt. In the Mobile App for Physical Activity intervention, for both cases, a communication from a credible source in favor of the active behavior was presented. In a final step, the selection was reviewed and confirmed by a panel of 4 senior researchers (Textbox 1).

It is important to note the limitations of the current literature in which the presented BCTs were identified. First, the Mobile App for Physical Activity intervention was developed for adolescents, although data from meta-analyses and reviews for adults were used as there is a gap concerning studies on mHealth PA promotion in younger populations [18]. Second, although the review by Brannon and Cushing [63] concentrates on apps, the meta-analysis they performed was based on *classic* PA interventions rather than apps. Third, the identified meta-analyses (also based on *classic* PA interventions) presented a mix of results in terms of BCTs. Therefore, we included BCTs in our list only if they were associated with PA in at least two of the 6 meta-analyses. Finally, yet importantly, there are several reviews on the topic published to date that can inform the reader on BCTs identified in efficacious interventions [11,69,70]; however, only 1 review (Sullivan et al [47]) provides specific recommendations on helpful strategies [47]. Therefore, we implemented the recommendations from these reviews and meta-analyses in that we coded all the BCTs presented according to previous versions of BCT taxonomies (26 and 40 BCTs) into the latest taxonomy (93 BCTs) [71-73].

The combination of potentially advantageous and attractive PA app features and the selection of BCTs associated with PA change supported a novel approach in developing the Mobile App for Physical Activity intervention. The selection of BCT components was based on two factors: (1) components were selected if considered attractive to adolescents and (2) components were selected if they were considered the most appropriate to reflect a certain BCT (eg, components such as *graphical representation of performed PA* would naturally include the BCT *Feedback on behavior*). The result (ie, the components of the app and the BCTs implemented in them) is presented in Table 1.

**Recommended, effective, and efficacious behavior change techniques (BCTs).**

**BCTs associated with physical activity (PA) identified in at least two of the 6 meta-analyses [51,63-67]**

- Self-monitoring of behaviour (2.3), self-monitoring of outcome(s) of behaviour (2.4)
- Goal setting (behaviour; 1.1), goal setting (outcome; 1.3)
- Feedback on behaviour (2.2), feedback on outcome(s) of behaviour (2.7)
- Information about health consequences (5.1)

**BCTs associated with PA according to the meta-analysis by Brannon and Cushing [63]**

- Information about health consequences (5.1)
- Information about others’ approval (6.3)
- Goal setting (behaviour; 1.1), goal setting (outcome; 1.3)
- Self-monitoring of behaviour (2.3), self-monitoring of outcome(s) of behaviour (2.4)
- Behavioural contract (1.8)

**BCTs recommended for implementation by Sullivan et al [47]**

- Goal setting: goal setting (behaviour; 1.1), goal setting (outcome; 1.3)
- Self-monitoring: self-monitoring of behaviour (2.3), self-monitoring of outcome(s) of behaviour (2.4)
- Feedback: feedback on behaviour (2.2), feedback on outcome(s) of behaviour (2.7)
- Rewards: reward (outcome; 10.10), nonspecific reward (10.3)
- Social support: social support (unspecified; 3.1), social support (practical; 3.2), social support (emotional; 3.3)
- Coaching: instruction on how to perform the behaviour (4.1)
- Identifying obstacles: problem solving (1.2)
- Restructuring negative attitudes: framing/reframing (13.2)
- Action planning: action planning (1.4)
- Modifying environmental factors: restructuring the physical environment (12.1)

**BCTs selected through the Behaviour Change Wheel framework** **[28,68]**

- Credible source (9.1)
- Monitoring of behaviour by others without feedback (2.1), monitoring of outcome(s) of behaviour without feedback (2.5)
- Adding objects to the environment (12.5)
- Review behaviour goal(s) (1.5), review outcome goal(s) (1.7)

**Table 1:** Behavior change techniques (BCTs) included in the Mobile App for Physical Activity intervention components.

Push or pull	Component	BCTs
Push	Personalized PA^a^ prompt	Feedback on behavior, prompts/cues, discrepancy between current behavior and goal, information about health consequences, credible source
Push	Reminders to move	Feedback on behavior, prompts/cues, discrepancy between current behavior and goal
Push	Rewards	Reward (outcome), self-monitoring of behavior
Pull	Interactive assistance	Problem solving, restructuring the physical environment, credible source
Pull	Graphs and stats	Goal setting (outcome), feedback on behavior, information about health consequences, monitoring of outcome(s) of behavior without feedback

^a^PA: physical activity.

### Theoretical Background

**Figure 1:**
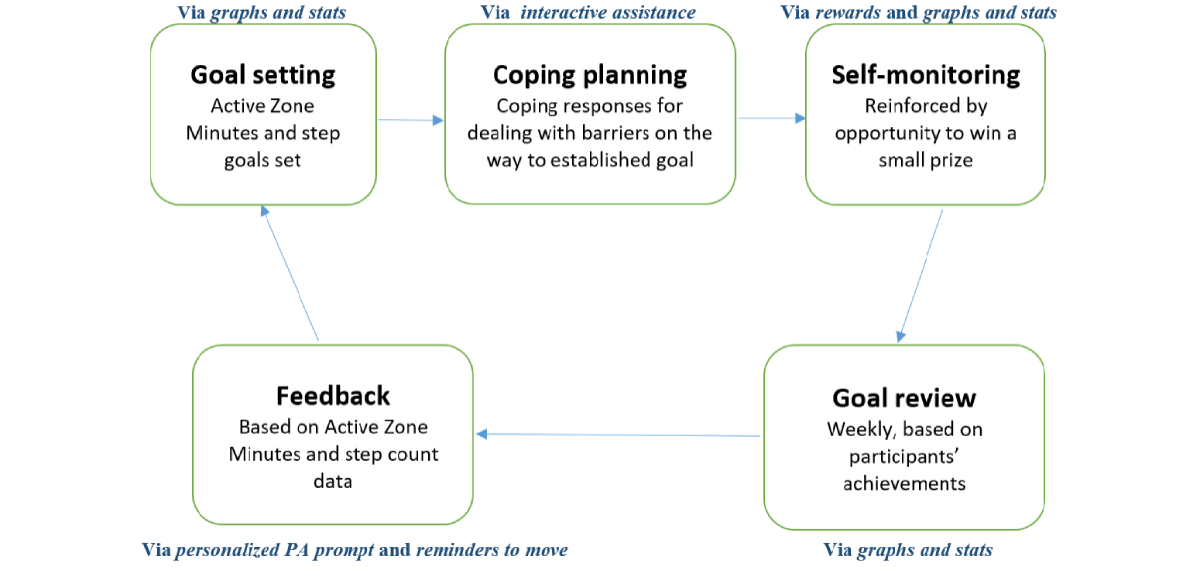
Components of adjusted self-regulation control theory, which informed the Mobile App for Physical Activity intervention. PA: physical activity.

A meta-regression by Michie et al [51] has demonstrated that including such BCTs as self-monitoring combined with at least one other BCT from control theory (ie, prompt intention formation, prompt specific goal setting, providing feedback on performance, prompt self-monitoring of behavior, and prompt review of behavioral goals) in PA interventions is effective [51]. Three of these BCTs—namely, self-monitoring, goal setting, and feedback—correspond to the process of self-regulation or, more specifically, control theory [74]. This theory suggests that self-monitoring behavior, receiving feedback, setting goals, and reviewing goals following feedback are central to behavioral self-management. Thus, the Mobile App for Physical Activity intervention components were centered on behavioral self-regulation (Figure 1). This, in addition to the aforementioned rigorous selection approach, enabled the selection and sequencing of the central BCTs [75].

### Participants

The selection criteria were similar to the ones applied in our focus group discussion study. A local international school was chosen to recruit healthy adolescents aged 16 to 18 years. The advertisement with study details was disseminated via the school’s email service, seeking to attract adolescents who were insufficiently active yet, on principle, willing to increase their PA participation. In addition, several adolescent students of local Luxembourgish schools were recruited from the participant list of the focus group discussion, which took place earlier and has been described elsewhere [76]. The advertisement contained general information about the study and informed potential participants that they would be remunerated for taking part. Remuneration was provided by letting the participants keep the activity tracker implemented in the study (Fitbit Charge 4) and the possibility to win sporting goods store vouchers. The participants needed to be fluent in English and possess a smartphone. Participants were excluded if they had any constraints toward performing PA and if they owned and actively used an activity tracker (eg, Fitbit, Garmin, or Apple Watch) as additional devices provided within the Mobile App for Physical Activity trial could be perceived as burdensome. The study participants were provided with an informed consent form, which had to be signed by the participants themselves (when aged 18 years) or their legal representative (when aged <18 years) before participation.

### Ethics Approval

This study was approved by the Ethics Review Panel of the University of Luxembourg (19-046A2 Mobile App for Physical Activity).

### Study Design and Procedure

The Mobile App for Physical Activity intervention was carried out as a 4-week within-subject trial (baseline week+3 intervention weeks). At the selection stage, the PA profile of the interested participants was evaluated using the Physical Activity Questionnaire for Adolescents (PAQ-A) [77]. This questionnaire was selected based on an expert panel ranking that evaluated the PAQ-A as one of the very few self-report instruments with acceptable reliability, practicality, and validity [78]. The PAQ-A is a 7-day-recall self-administered questionnaire designed to provide a general estimate of PA levels in healthy adolescents aged between 8 and 20 years derived from a series of questionnaire items on activity during and after school, sports participation, and activity in the evenings and weekends [77]. Participants were included if they had a PAQ-A score of ≤3 out of 5 (low to moderate levels of weekly PA). An introduction session was organized for the participants at intake to provide them with general information; create Fitbit accounts; link the fitness trackers to them; and analyze their PA habits, basic sociodemographic information, and previous experiences with PA and fitness apps. The participants were then given activity trackers and instructed to wear them at all times (including bathing, sleeping, and swimming). The participants were also instructed to install the Fitbit app and create a Fitbit account while all the notifications for the Fitbit app (and activity tracker) were disabled during the baseline week, and users were encouraged to ignore any notifications that might appear erroneously. Credentials for the Fitbit account were shared with AD to further turn on certain notifications and features during the treatment weeks. The participants were also asked not to use the Fitbit app during the baseline week. AD also connected with all the participants via the Fitbit app (*Add friends* feature) to send them later the personalized PA prompt via the dedicated *Messages* chat tab in the Fitbit app. After the initial week (a baseline period), notifications were turned on remotely via Fitbit account manipulation for proper functioning of components, and personalized step and Active Zone Minute goals were set. Users were informed that the Fitbit app could be assessed at any time without a prerequired frequency. Adherence to both the Fitbit app and the activity tracker and data collection was monitored through the Fitabase platform [79]. Emails were sent to the participants when tracker data were missing for >24 hours or when the participants forgot to synchronize data. If data were not received after 48 hours, the participant was contacted by the study supervisor via SMS text message or a phone call. Normally, this situation did not occur more often than 4 to 6 times per week.

### Statistical Analysis

The aggregated data set was downloaded in CSV format from the Fitbit application programming interface via the Fitabase platform. Fitbit provides data of a different resolution (days or minutes) and, for the primary analysis, we aggregated daily into weekly records.

To test the first and second hypotheses, we [80] performed paired 1-tailed *t* tests to reveal within-subject differences in the primary and secondary outcome measures between measurement occasions (baseline, week 1, week 2, and week 3). We tested for the outcome measures outlined in Textbox 2.

**Primary and secondary outcome measures.**

**Primary outcome**

- Sedentary minutes based on the metabolic equivalent of task (MET)

**Secondary outcomes**

- Active Minutes based on MET or moderate to vigorous physical activity (MVPA) minutes
- Active Zone Minutes based on heart rate or MVPA minutes
- Step count

As the 2 recruitment sources (international school and local schools) had different holiday schedules, we plotted for differences in outcomes between participants from these 2 groups. Owing to the small sample size, we computed a bootstrapped paired 1-tailed *t* test in addition to the classic paired 1-tailed *t* test. The measurement occasions were 7-day periods, further referred to as *baseline*, *week 1*, *week 2*, and *week 3*. Each week 1 to week 3 value was compared with the baseline. The α level was set to .05 for all tests. While α inflation is an issue with multiple testing, we decided not to use the Bonferroni correction as it is considered overly conservative and, therefore, increases the risk of type 2 error. Given the early stage of development and research in this area, we would argue that reporting the uncorrected results contributes to the literature by stimulating studies with larger samples in which hypotheses can then be tested more rigorously.

To test the third hypothesis, we performed change-point detection analysis to identify change points in the means of the step count and the HR time series in the minute-to-minute resolution. As personalized PA prompts were given every day between 5 PM and 7 PM, we used a time frame of 3 PM to 7 PM to estimate change points. Once estimated, we chose the best-fitting change point after 5 PM per participant and day. We then subtracted the averaged step counts and HR of the 30-minute period before from the 30-minute period after the change point to calculate the magnitude of physiological change at that time. The 30-minute period was chosen based on the approach by Klasnja et al [24]. As a 30-minute period value was rather theory-inspired, we used an empirical approach and tested for the magnitude of physiological change also within a 60-minute period. To analyze the differences between weeks, we aggregated the magnitude of HR changes per participant and week. Although we did not provide a personalized PA prompt during the baseline, we nevertheless controlled for the random variations from 5 PM to 7 PM, which resulted in a comparison value for the treatment weeks. All statistical analyses were carried out using RStudio (version 4.1.1; R Foundation for Statistical Computing) [80].

## Results

### Sample

We recruited 18 participants, of whom 6 (33%) were women. Most participants (11/18, 61%) were recruited from an international school, whereas the other 39% (7/18) were recruited from Luxembourgish schools. The participants had different levels of motivation to improve their PA behavior, varying from very low to moderate. For most of the participants (11/18, 61%)), English was their primary language. Nevertheless, plain English was used within the Mobile App for Physical Activity intervention to make it more attractive to all the participants. The participant mean age was 16.33 (SD 0.57) years. All participants (18/18, 100%) filled in the PAQ-A questionnaire, ranging from a score of 1, which indicates a low PA level, to a score of 5, which indicates a high PA level, with a mean score of 2.72 (SD 0.48). Most participants (16/18, 89%) owned an iOS-powered smartphone, with 11% (2/18) of the participants owning an Android-powered smartphone. We conducted the analysis while differentiating (color coding) between participants from the international school and Luxembourgish schools. However, the recruitment site was not introduced as a between-subject factor owing to the small sample size; therefore, we did not further report data on the differences between recruitment sites, describing rather our general observations of the differences between the 2 sites. Although showing similar trends in sedentary behavior, the step count and MVPA minute trends were generally divergent; students from local schools tended to increase their step count and MVPA minutes, whereas students from the international school tended to decrease their step count and MVPA minutes over the trial period.

### Primary Outcome Analysis: Change in Daily Time Spent in Sedentary Behavior Based on MET

**Figure 2:**
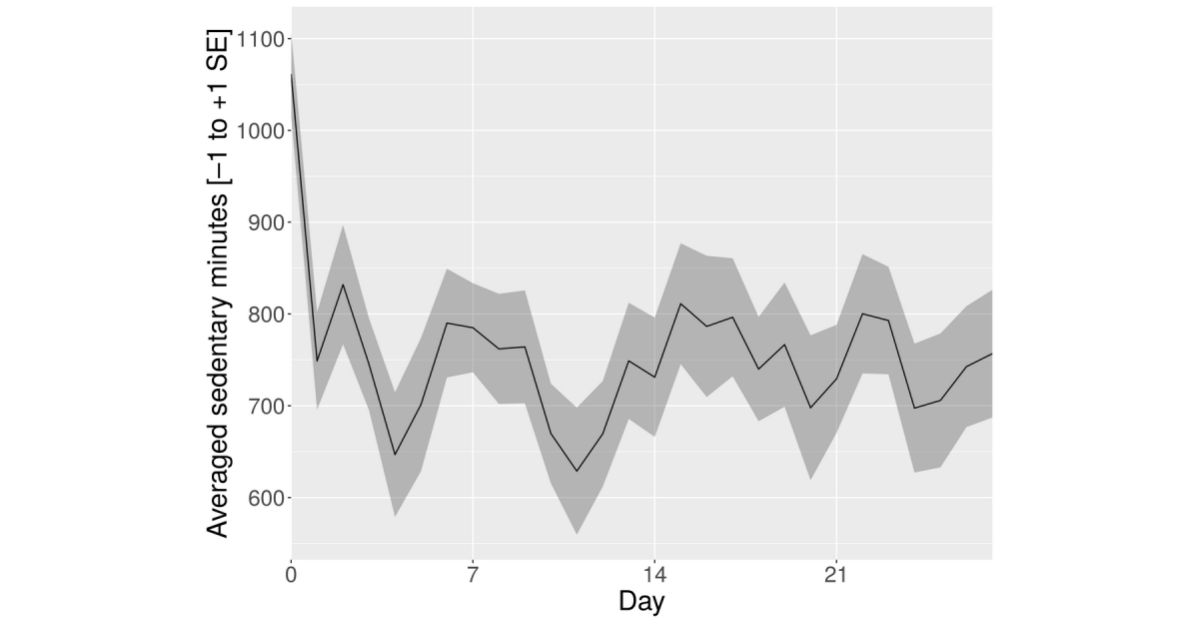
Averaged sedentary minutes (mean+SE of the mean).

Sedentary minute counts decreased significantly during the first week of the trial compared with the baseline (*t*_17_=−1.79; *P*=.04; bootstrapped *P*=.02; Table 2). This effect diminished over time and was no longer significant at week 2 (*t*_17_=−0.51; *P*=.30; bootstrapped *P*=.30) and week 3 (*t*_17_=−0.94; *P*=.17; bootstrapped *P*=.21). Figure 2 depicts the time course of sedentary minutes over the entire duration of the intervention. The error area in this and the other figures represents the SE of the mean.

**Table 2:** Outcome measures during the baseline and intervention periods.

Outcome and measurement occasion			Values per day, mean (SD)
**Sedentary behavior (minutes per week)**			
	Baseline	789.34 (176.71)	
	Week 1	718.30 (177.58)	
	Week 2	764.09 (209.89)	
	Week 3	749.13 (209.80)	
**MVPA** ^a^ **based on MET^b^ (minutes per week)**			
	Baseline	90.37 (47.94)	
	Week 1	100.85 (46.24)	
	Week 2	95.50 (55.08)	
	Week 3	91.57 (49.10)	
**MVPA based on HR** ^c^ **(minutes per week)**			
	Baseline	42.88 (27.41)	
	Week 1	45.88 (27.15)	
	Week 2	37.81 (24.51)	
	Week 3	39.94 (32.66)	
**Step count (steps per week)**			
	Baseline	14,134.56 (4980.45)	
	Week 1	14,298.28 (4523.17)	
	Week 2	13,581.28 (5529.25)	
	Week 3	14,126.29 (5413.41)	

^a^MVPA: moderate to vigorous physical activity.

^b^MET: metabolic equivalent of task.

^c^HR: heart rate.

### Secondary Outcome Analyses

#### Change in MVPA Minutes Based on MET

**Figure 3:**
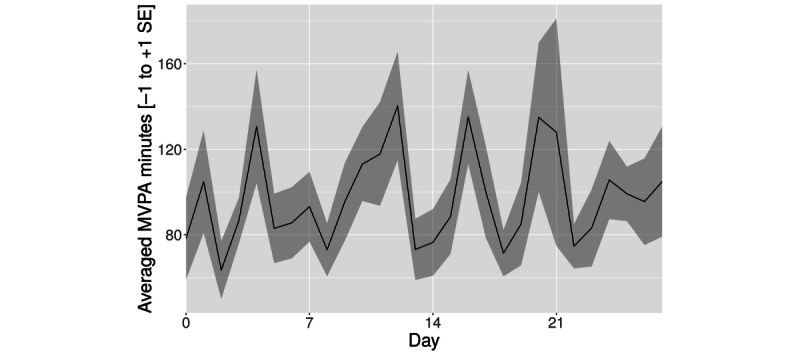
Averaged moderate to vigorous physical activity (MVPA) minutes based on the metabolic equivalent of task (mean+SE of the mean).

In total, 2 outliers with >500 MVPA minutes a day were excluded. With MET-based MVPA minutes, we observed a reversed nonsignificant trend in comparison with sedentary minute count (Table 2)—in the first week, the descriptive values of MET-based MVPA minutes increased compared with the baseline (*t*_17_=1.23; *P*=.11; bootstrapped *P*=.12). This effect diminished over time and, although it was still positive in week 2 (*t*_17_=0.41; *P*=.34; bootstrapped *P*=.34), it was smaller in week 3 (*t*_17_=0.12; *P*=.45; bootstrapped *P*=.45). None of these changes reached significance levels (see Figure 3 for the complete time course).

#### Change in MVPA Minutes Based on HR

**Figure 4:**
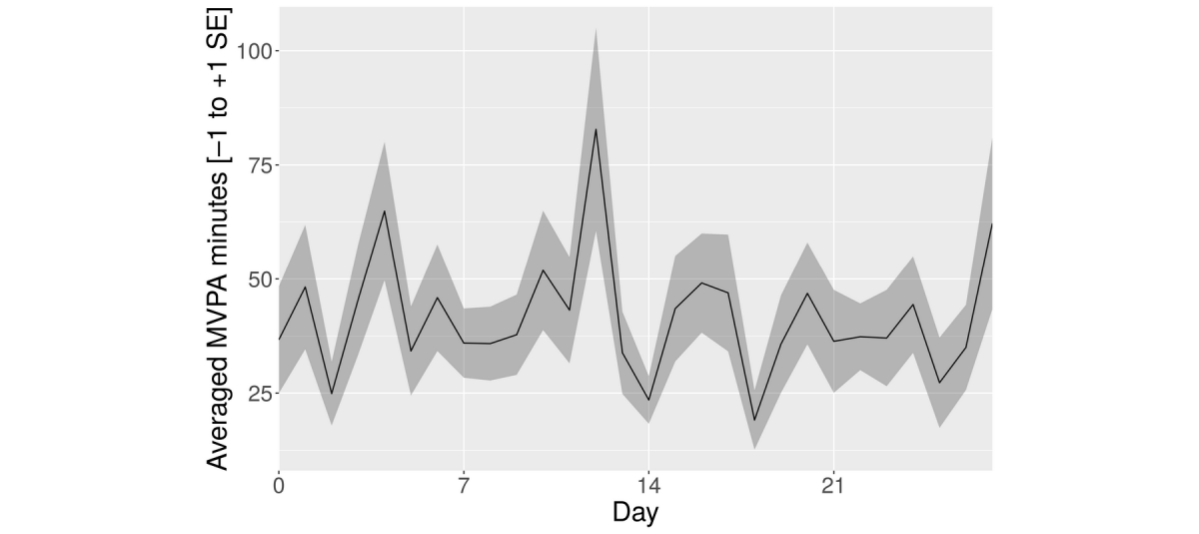
Averaged moderate to vigorous physical activity (MVPA) minutes based on heart rate (mean+SE of the mean).

Although we observed an initial nonsignificant increase in the first week (*t*_17_=0.50; *P*=.31; bootstrapped *P*=.29), we observed a nonsignificant decline compared with the baseline for weeks 2 (*t*_17_=−0.57; *P*=.71; bootstrapped *P*=.72) and 3 (*t*_17_=−0.39; *P*=.65; bootstrapped *P*=.63). See Figure 4 for the complete time course.

#### Change in Step Count

**Figure 5:**
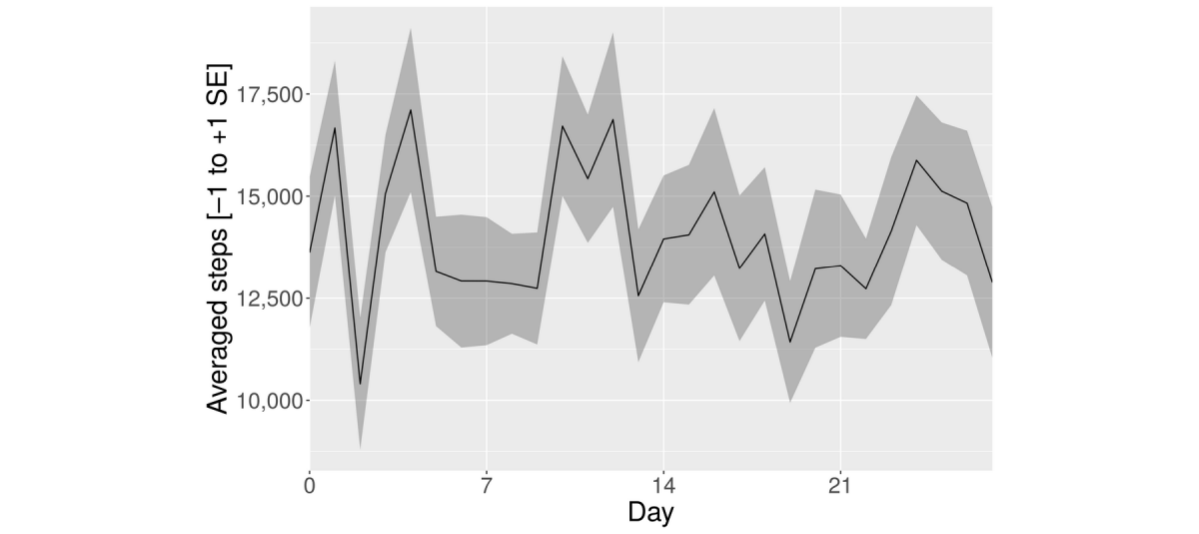
Averaged steps (mean+SE of the mean).

The analysis of step count revealed a descriptive course similar to the results of the MVPA minutes based on HR (Table 2)—although we observed a slight nonsignificant increase in the first week (*t*_17_=0.20; *P*=.41; bootstrapped *P*=.43), there was no clear linear trend over the following weeks. In weeks 2 (*t*_17_=−0.33; *P*=.62; bootstrapped *P*=.63) and 3 (*t*_17_=−0.006; *P*=.50; bootstrapped *P*=.50), we observed a nonsignificant decline compared with the baseline (Figure 5).

### Estimation of the Effect After the Delivery of a Personalized PA Prompt: Change-Point Analyses

#### HR at 60 Minutes

**Figure 6:**
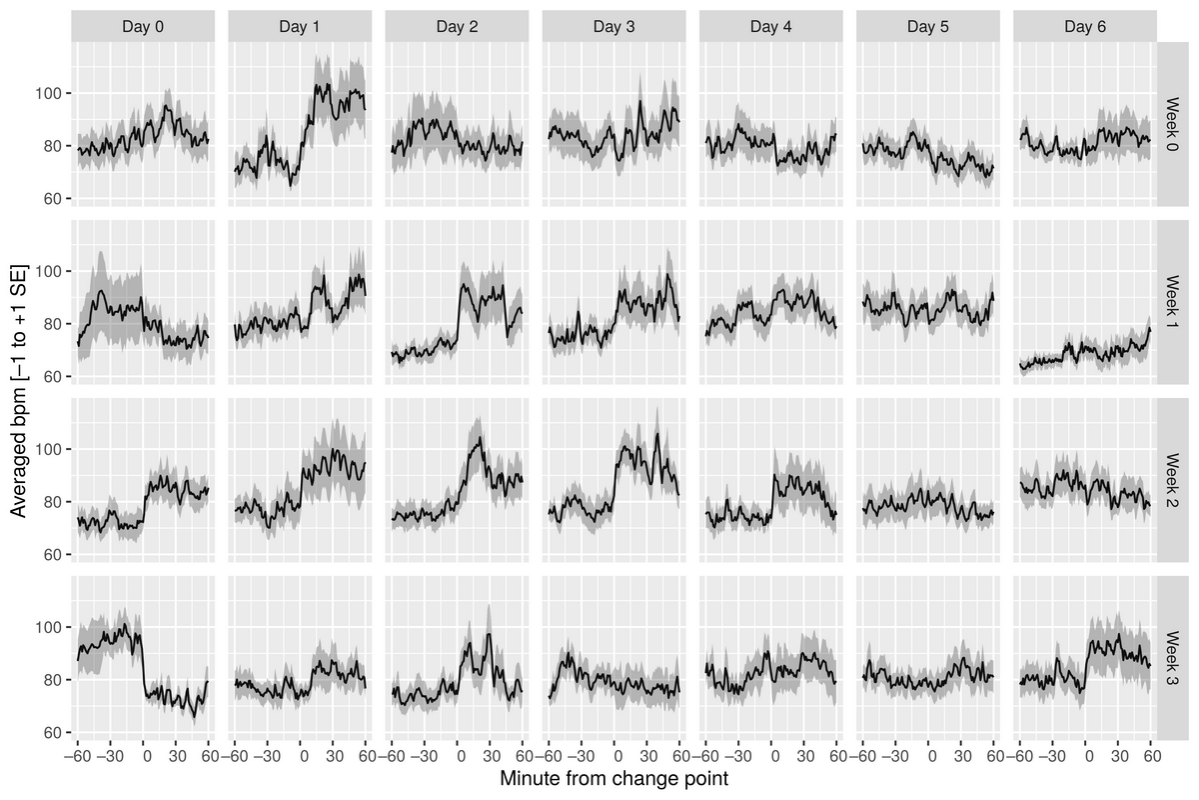
Change-point analysis: heart rate (mean+SE of the mean). bpm: beats per minute.

We linearly interpolated some missing data (up to 5 minutes per day) to be able to perform the change-point analysis. If >5 minutes per day were missing, data from that day were excluded before running the change-point analysis. As shown in Table 3, the intervention resulted in a nonsignificant increase in HR compared with the baseline in the first week (*t*_16_=1.28; *P*=.10; bootstrapped *P*=.09) and a significant increase in the second week (*t*_16_=1.84; *P*=.04; bootstrapped *P*=.04). However, this effect diminished during week 3 (*t*_16_=−0.07; *P*=.52; bootstrapped *P*=.52), where we observed a nonsignificant decline compared with the baseline (see Figure 6 for the complete time course).

**Table 3:** Change-point analysis results.

Outcome and measurement occasion			Minutes before		Minutes after		Increase per minute, mean (SD)
**Change-point analysis: heart rate at 60 minutes (bpm)**							
	Baseline	80.637		81.762		1.12 (11.09)	
	Week 1	77.617		84.211		6.59 (11.73)	
	Week 2	78.877		89.024		10.14 (16.23)	
	Week 3	83.183		80.831		−2.35 (17.29)	
**Change-point analysis: heart rate at 30 minutes (bpm)**							
	Baseline	81.256		81.893		0.63 (13.28)	
	Week 1	78.656		84.534		5.87 (10.36)	
	Week 2	79.496		90.772		11.27 (15.88)	
	Week 3	83.647		81.865		−1.78 (15.74)	
**Change-point analysis: step count at 60 minutes (steps)**							
	Baseline	16.955		23.604		6.64 (13.10)	
	Week 1	19.813		25.510		5.69 (17.28)	
	Week 2	13.912		27.997		14.08 (21.92)	
	Week 3	17.826		23.755		5.92 (12.70)	
**Change-point analysis: step count at 30 minutes (steps)**							
	Baseline	19.732		27.038		7.30 (16.18)	
	Week 1	20.191		29.808		9.61 (16.50)	
	Week 2	15.036		30.855		15.81 (22.52)	
	Week 3	19.794		26.224		6.42 (13.33)	

#### HR at 30 Minutes

The intervention resulted in a nonsignificant increase in HR compared with the baseline in the first week (*t*_16_=1.15; *P*=.13; bootstrapped *P*=.13) and a significant increase in the second week (*t*_16_=1.95; *P*=.03; bootstrapped *P*=.03). However, this effect diminished during week 3 (*t*_16_=0.07; *P*=.47; bootstrapped *P*=.46), where we observed a nonsignificant decline compared with the baseline (Figure 6).

#### Step Count at 60 Minutes

**Figure 7:**
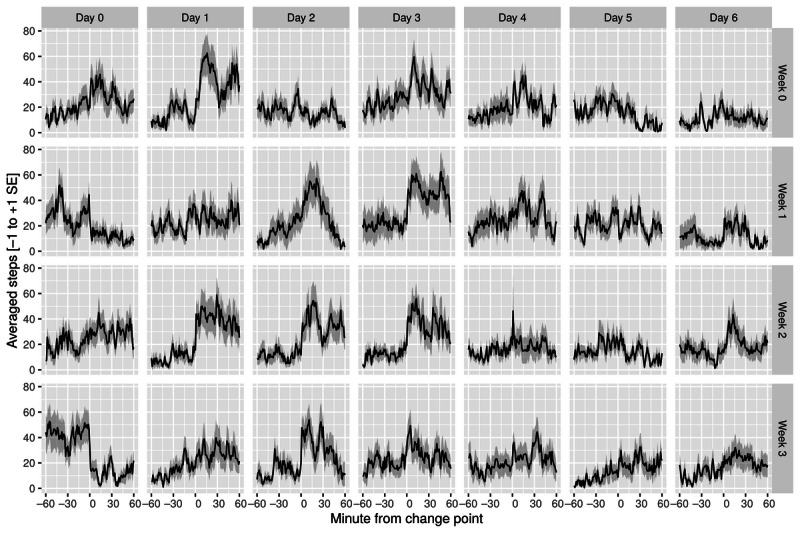
Change-point analysis: steps (mean+SE of the mean).

Change-point analysis of step count revealed no clear linear trend (Table 3)—although we observed an insignificant decrease in the first week (*t*_17_=−0.23; *P*=.59; bootstrapped *P*=.58), there was a significant increase in the second week (*t*_17_=1.35; *P*=.09; bootstrapped *P*=.05). In week 3 (*t*_17_=−0.21; *P*=.58; bootstrapped *P*=.56), we observed a nonsignificant decline compared with the baseline (see Figure 7 for the complete time course).

#### Step Count at 30 Minutes

As shown in Table 3, the change-point analysis of step count revealed a trend similar to the 60-minute measurement period—although we observed an insignificant increase in the first week (*t*_17_=0.50; *P*=.30; bootstrapped *P*=.29), there was an increase in the second week, which was significant (*t*_17_=1.34; *P*=.09; bootstrapped *P*=.05). In week 3 (*t*_17_=−0.22; *P*=.58; bootstrapped *P*=.58), we observed a nonsignificant decline compared with the baseline (Figure 7).

## Discussion

### Principal Findings

This study, to our knowledge, is the first to develop a behavior change, theory-informed PA mHealth intervention with personalized prompts for adolescents aged 16 to 18 years evaluated using a within-subject experimental design. In contrast to the widespread 1D approach (eg, step count only [12,24,38,40]), this study involved the inclusion of 4 outcome measures to assess the multidimensional PA user profiles.

Overall, the results showed that the Mobile App for Physical Activity smartphone-based intervention produced significant reductions in sedentary time among adolescents during the first week of the trial. This trend, although it remained positive, diminished over time. This may be related to several reasons, including the holiday period, or certain aspects of the intervention being perceived as burdensome. This suggests that the implementation of the Mobile App for Physical Activity intervention may result in better health outcomes for adolescents, although there is currently insufficient evidence available to determine a specific dose-response relationship between sedentary time and health outcomes in adolescents [81]. Our findings indicate that the intervention had no effect on MET-based MVPA minutes, although the descriptive increase may give reason for further investigation. Although the results suggested no overall change in HR-based MVPA minutes, the results from the change-point analyses suggest that the personalized PA prompts significantly increased HR per minute (bpm) during the second week of the study. There were no significant increases in the participants’ overall step count; however, the personalized PA prompts resulted in a marginally significant increase in step counts per minute in the second week of the study. The results also revealed that the participants’ engagement, based on the amount of missing data and responses to app suggestions, although initially high, decreased over the study period. These results may suggest that the intervention was successful in giving adolescents a nudge strong enough to interrupt and decrease their sedentary behavior but insufficient for a more high-effort increase in MVPA minutes. Personalized PA prompts, although moderately successful in promoting a light activity increase, did not result in a more intense MVPA minute increase.

As noted in the *Results* section, there were noticeable differences between participants from the 2 recruitment sites. This study was carried out between the summer term and the vacation period; therefore, this divergence may be explained by the fact that the 2 schools differed in their holiday calendars. Specifically, although the holiday period for students from Luxembourgish schools started at week 3 of the study, students from the international school were in the holiday period earlier, from week 1 onward. This may have resulted in an earlier decrease in PA for students from the international school compared with students from Luxembourgish schools. The descriptive lack of correlation between step count levels and MVPA levels confirmed the importance of accounting for various outcome measures while tracking the participants’ PA in several dimensions.

Our study supports the results of a previous study by Bond et al [25], where a smartphone-based intervention yielded significant decreases in MET-based sedentary behavior in adults, which may confirm that smartphone-based PA interventions also have a high potential among adolescent populations. These results are also in line with some of the previous findings of the studies by Rabbi et al [23] and Klasnja et al [24] supporting the beneficial impact of personalized PA suggestions for adolescents. These results partially confirm the findings of the study by Kramer et al [82] supporting the use of financial incentives to initiate increased PA. However, future interventions should consider the exit strategy where, in time, participants would sustain increased PA levels based on intrinsic rather than extrinsic motivation. Challenges concerning the limited engagement of adolescents (based on the amount of missing data and decreased response to app suggestions over time) were similar to problems encountered by Lubans et al [83]. Engagement with mHealth PA interventions remains an important challenge to overcome for behavior change experts and developers in future interventions. Finally, our findings partially confirm the findings of the study by Gaudet et al [27], in which a minimalistic intervention based on the Fitbit activity tracker resulted in MVPA minute increases in adolescents, which may suggest that interventions including commercial fitness trackers may be advantageous for interventions among adolescent populations. Most published smartphone-based intervention studies such as ours include a relatively small participant sample. Therefore, it is important for future studies to replicate these findings and extend them to larger samples to further investigate approaches to increase adolescents’ PA.

This study used a set of devices and a data platform that are designed to improve sedentary behavior and PA levels among adolescents and are currently commercially available. With their accessibility and relatively low price, compact and waterproof HR- and GPS-powered wrist-worn devices [31] in combination with research-grade data collection platforms provide researchers with attractive solutions for data collection and analysis, mitigating burdensomeness and intervention development time span.

A small number of studies on adolescents in the domain of mHealth PA and even fewer studies using theory-informed interventions call for future research in this area to further knowledge accumulation, both qualitative and quantitative. Systematic methods of intervention development with the help of tools such as the Behaviour Change Wheel and the BCT Taxonomy should be applied further by researchers to allow for the identification of effective intervention components and BCTs for the adolescent age group. Further sustainability of PA and sedentary behavior changes should be investigated via longitudinal studies. Finally, future research should implement alternative designs such as a within-subject design or MRT, which may investigate the efficacy of the intervention’s individual components within a relatively short study duration.

These results suggest the feasibility and promise of smartphone-based PA interventions with personalized PA suggestions for adolescents. Although minimalist in nature, the introduction of such an intervention may represent a sufficient trigger for adolescents to decrease their sedentary behavior and increase their PA levels.

### Strengths and Limitations

This study has several strengths. It is one of the few studies to develop and test an mHealth PA intervention for adolescents. Key methodological strengths include (1) the multidimensional PA profile assessment, specifically using versatile outcome measures; (2) the rigorous multistage theoretical development of the intervention guided by intervention development frameworks, taxonomies, and the latest research findings; and (3) the use of the latest wearable device and data collection platform, which presented inherent advantages and features, including undemanding data collection, quick device acceptance by participants, and prompt feedback time between participants and researchers.

This study also has important limitations. First, probably aggravated by restrictions imposed by the COVID-19 pandemic, we managed to enroll only a relatively small participant sample. Second, some participants did not wear the device for the entire duration of the study, taking it off during sleep or certain activities as wearing a watch or a fitness tracker was considered dangerous; for instance, in martial arts classes. The participants also forgot to wear the device on several occasions after sleep or forgot to charge the device in a timely manner, which resulted in missing data. Another important limitation is the short-term and small-scale nature of this study, which reduces the possibility to come to exhaustive conclusions. It is also important to note that the participants took part in the study during the summer holiday period, which may have affected their PA patterns. In line with previous studies, during the baseline week of the study, we turned all the notifications off. However, we could not fully ensure that the participants would not use a Fitbit app or check the data provided by the fitness tracker either; similarly, we could not ascertain that the notifications during the treatment weeks would be read at once. The participants’ second-language proficiency may have affected their overall engagement with the intervention. Finally, as the proprietary algorithms used to calculate HR- and MET-based MVPA minutes are not publicly available, caution must be taken when interpreting PA data collected by such trackers.

Despite these limitations, this study provides preliminary evidence of the usefulness of an mHealth PA smartphone intervention while shedding light on potential directions for future mHealth PA smartphone intervention developments.

### Conclusions

This study provides preliminary evidence of the benefits of the Mobile App for Physical Activity intervention for modest yet significant reductions in the participants’ sedentary time and the beneficial role of personalized PA prompts. These results also provide further evidence of the benefits and relative efficacy of personalized activity suggestions for inclusion in smartphone-based PA interventions. This study also provides an example of how to guide the development of subsequent smartphone-based mHealth PA interventions for adolescents. Future investigations should focus on replicating these findings and testing the potential for scalability of such an intervention in larger population samples.

## Abbreviations

BCT: behavior change technique
HR: heart rate
JITAI: just-in-time adaptive intervention
MET: metabolic equivalent of task
mHealth: mobile health
MRT: microrandomized trial
MVPA: moderate to vigorous physical activity
PA: physical activity
PAQ-A: Physical Activity Questionnaire for Adolescents
RCT: randomized controlled trial
WHO: World Health Organization

## References

[1] (2016). Health at a Glance: Europe 2016 – State of Health in the EU Cycle. Organisation for Economic Co-operation and Development, European Union.

[2] Lee IM, Shiroma EJ, Lobelo F, Puska P, Blair SN, Katzmarzyk PT, Lancet Physical Activity Series Working Group (2012). Effect of physical inactivity on major non-communicable diseases worldwide: an analysis of burden of disease and life expectancy. Lancet.

[3] (2020). Health at a Glance: Europe 2020 – State of Health in the EU Cycle. Organisation for Economic Co-operation and Development, European Union.

[4] (2010). A National Survey of Children and Young People’s Physical Activity and Dietary Behaviours in New Zealand: 2008/09. National Institute for Health Innovation.

[5] Patrick K, Griswold WG, Raab F, Intille SS (2008). Health and the mobile phone. Am J Prev Med.

[6] Klasnja P, Hekler EB, Shiffman S, Boruvka A, Almirall D, Tewari A, Murphy SA (2015). Microrandomized trials: an experimental design for developing just-in-time adaptive interventions. Health Psychol.

[7] Nahum-Shani I, Smith SN, Spring BJ, Collins LM, Witkiewitz K, Tewari A, Murphy SA (2018). Just-in-Time Adaptive Interventions (JITAIs) in mobile health: key components and design principles for ongoing health behavior support. Ann Behav Med.

[8] Hekler EB, Buman MP, Otten J, Castro CM, Grieco L, Marcus B, Friedman RH, Napolitano MA, King AC (2013). Determining who responds better to a computer- vs. human-delivered physical activity intervention: results from the community health advice by telephone (CHAT) trial. Int J Behav Nutr Phys Act.

[9] King AC, Hekler EB, Castro CM, Buman MP, Marcus BH, Friedman RH, Napolitano MA (2014). Exercise advice by humans versus computers: maintenance effects at 18 months. Health Psychol.

[10] King AC, Hekler EB, Grieco LA, Winter SJ, Sheats JL, Buman MP, Banerjee B, Robinson TN, Cirimele J (2013). Harnessing different motivational frames via mobile phones to promote daily physical activity and reduce sedentary behavior in aging adults. PLoS One.

[11] Schoeppe S, Alley S, Van Lippevelde W, Bray NA, Williams SL, Duncan MJ, Vandelanotte C (2016). Efficacy of interventions that use apps to improve diet, physical activity and sedentary behaviour: a systematic review. Int J Behav Nutr Phys Act.

[12] Walsh JC, Corbett T, Hogan M, Duggan J, McNamara A (2016). An mHealth intervention using a smartphone app to increase walking behavior in young adults: a pilot study. JMIR Mhealth Uhealth.

[13] Wang L, Miller LC (2020). Just-in-the-Moment Adaptive Interventions (JITAI): a meta-analytical review. Health Commun.

[14] Hardeman W, Houghton J, Lane K, Jones A, Naughton F (2019). A systematic review of just-in-time adaptive interventions (JITAIs) to promote physical activity. Int J Behav Nutr Phys Act.

[15] Knight E, Stuckey MI, Prapavessis H, Petrella RJ (2015). Public health guidelines for physical activity: is there an app for that? A review of android and apple app stores. JMIR Mhealth Uhealth.

[16] Stuckey MI, Carter SW, Knight E (2017). The role of smartphones in encouraging physical activity in adults. Int J Gen Med.

[17] Michie S, Carey RN, Johnston M, Rothman AJ, de Bruin M, Kelly MP, Connell LE (2018). From theory-inspired to theory-based interventions: a protocol for developing and testing a methodology for linking behaviour change techniques to theoretical mechanisms of action. Ann Behav Med.

[18] Domin A, Spruijt-Metz D, Theisen D, Ouzzahra Y, Vögele C (2021). Smartphone-based interventions for physical activity promotion: scoping review of the evidence over the last 10 years. JMIR Mhealth Uhealth.

[19] McCallum C, Rooksby J, Gray CM (2018). Evaluating the impact of physical activity apps and wearables: interdisciplinary review. JMIR Mhealth Uhealth.

[20] Pham Q, Wiljer D, Cafazzo JA (2016). Beyond the randomized controlled trial: a review of alternatives in mHealth clinical trial methods. JMIR Mhealth Uhealth.

[21] Kumar S, Nilsen WJ, Abernethy A, Atienza A, Patrick K, Pavel M, Riley WT, Shar A, Spring B, Spruijt-Metz D, Hedeker D, Honavar V, Kravitz R, Lefebvre RC, Mohr DC, Murphy SA, Quinn C, Shusterman V, Swendeman D (2013). Mobile health technology evaluation: the mHealth evidence workshop. Am J Prev Med.

[22] Riley WT, Glasgow RE, Etheredge L, Abernethy AP (2013). Rapid, responsive, relevant (R3) research: a call for a rapid learning health research enterprise. Clin Transl Med.

[23] Rabbi M, Pfammatter A, Zhang M, Spring B, Choudhury T (2015). Automated personalized feedback for physical activity and dietary behavior change with mobile phones: a randomized controlled trial on adults. JMIR Mhealth Uhealth.

[24] Klasnja P, Smith S, Seewald NJ, Lee A, Hall K, Luers B, Hekler EB, Murphy SA (2019). Efficacy of contextually tailored suggestions for physical activity: a micro-randomized optimization trial of HeartSteps. Ann Behav Med.

[25] Bond DS, Thomas JG, Raynor HA, Moon J, Sieling J, Trautvetter J, Leblond T, Wing RR (2014). B-MOBILE--a smartphone-based intervention to reduce sedentary time in overweight/obese individuals: a within-subjects experimental trial. PLoS One.

[26] Kramer JN, Künzler F, Mishra V, Presset B, Kotz D, Smith S, Scholz U, Kowatsch T (2019). Investigating intervention components and exploring states of receptivity for a smartphone app to promote physical activity: protocol of a microrandomized trial. JMIR Res Protoc.

[27] Gaudet J, Gallant F, Bélanger M (2017). A bit of fit: minimalist intervention in adolescents based on a physical activity tracker. JMIR Mhealth Uhealth.

[28] Michie S, Atkins L, West R (2014). The Behaviour Change Wheel: A Guide To Designing Interventions.

[29] Tudor-Locke C, Craig CL, Beets MW, Belton S, Cardon GM, Duncan S, Hatano Y, Lubans DR, Olds TS, Raustorp A, Rowe DA, Spence JC, Tanaka S, Blair SN (2011). How many steps/day are enough? For children and adolescents. Int J Behav Nutr Phys Act.

[30] (2021). Fitbit application. Google Play.

[31] (2021). Fitbit Charge 4. Fitbit Inc.

[32] Thompson D, Peacock O, Western M, Batterham AM (2015). Multidimensional physical activity: an opportunity, not a problem. Exerc Sport Sci Rev.

[33] (2020). Actigraph device comparison. ActiGraph.

[34] Evenson KR, Goto MM, Furberg RD (2015). Systematic review of the validity and reliability of consumer-wearable activity trackers. Int J Behav Nutr Phys Act.

[35] Straiton N, Alharbi M, Bauman A, Neubeck L, Gullick J, Bhindi R, Gallagher R (2018). The validity and reliability of consumer-grade activity trackers in older, community-dwelling adults: a systematic review. Maturitas.

[36] Choi J, Lee JH, Vittinghoff E, Fukuoka Y (2016). mHealth physical activity intervention: a randomized pilot study in physically inactive pregnant women. Matern Child Health J.

[37] Duncan MJ, Vandelanotte C, Trost SG, Rebar AL, Rogers N, Burton NW, Murawski B, Rayward A, Fenton S, Brown WJ (2016). Balanced: a randomised trial examining the efficacy of two self-monitoring methods for an app-based multi-behaviour intervention to improve physical activity, sitting and sleep in adults. BMC Public Health.

[38] Korinek EV, Phatak SS, Martin CA, Freigoun MT, Rivera DE, Adams MA, Klasnja P, Buman MP, Hekler EB (2018). Adaptive step goals and rewards: a longitudinal growth model of daily steps for a smartphone-based walking intervention. J Behav Med.

[39] Middelweerd A, Te Velde SJ, Mollee JS, Klein MC, Brug J (2018). App-based intervention combining evidence-based behavior change techniques with a model-based reasoning system to promote physical activity among young adults (Active2Gether): descriptive study of the development and content. JMIR Res Protoc.

[40] van Woudenberg TJ, Bevelander KE, Burk WJ, Smit CR, Buijs L, Buijzen M (2018). A randomized controlled trial testing a social network intervention to promote physical activity among adolescents. BMC Public Health.

[41] Fuller D, Colwell E, Low J, Orychock K, Tobin MA, Simango B, Buote R, Van Heerden D, Luan H, Cullen K, Slade L, Taylor NG (2020). Reliability and validity of commercially available wearable devices for measuring steps, energy expenditure, and heart rate: systematic review. JMIR Mhealth Uhealth.

[42] Brewer W, Swanson BT, Ortiz A (2017). Validity of Fitbit's active minutes as compared with a research-grade accelerometer and self-reported measures. BMJ Open Sport Exerc Med.

[43] Dorn D, Gorzelitz J, Gangnon R, Bell D, Koltyn K, Cadmus-Bertram L (2019). Automatic identification of physical activity type and duration by wearable activity trackers: a validation study. JMIR Mhealth Uhealth.

[44] Nazari G, MacDermid JC, Sinden KE, Richardson J, Tang A (2019). Inter-instrument reliability and agreement of Fitbit charge measurements of heart rate and activity at rest, during the modified Canadian aerobic fitness test, and in recovery. Physiother Can.

[45] Degroote L, De Paepe A, De Bourdeaudhuij I, Van Dyck D, Crombez G (2021). Effectiveness of the mHealth intervention 'MyDayPlan' to increase physical activity: an aggregated single case approach. Int J Behav Nutr Phys Act.

[46] King AC, Campero I, Sheats JL, Castro Sweet CM, Garcia D, Chazaro A, Blanco G, Hauser M, Fierros F, Ahn DK, Diaz J, Done M, Fernandez J, Bickmore T (2017). Testing the comparative effects of physical activity advice by humans vs. computers in underserved populations: the COMPASS trial design, methods, and baseline characteristics. Contemp Clin Trials.

[47] Sullivan AN, Lachman ME (2017). Behavior change with fitness technology in sedentary adults: a review of the evidence for increasing physical activity. Front Public Health.

[48] Sniehotta FF, Schwarzer R, Scholz U, Schüz B (2005). Action planning and coping planning for long-term lifestyle change: theory and assessment. Eur J Soc Psychol.

[49] Bidargaddi N, Almirall D, Murphy S, Nahum-Shani I, Kovalcik M, Pituch T, Maaieh H, Strecher V (2018). To prompt or not to prompt? A microrandomized trial of time-varying push notifications to increase proximal engagement with a mobile health app. JMIR Mhealth Uhealth.

[50] (2020). WHO guidelines on physical activity and sedentary behaviour. World Health Organization.

[51] Michie S, Abraham C, Whittington C, McAteer J, Gupta S (2009). Effective techniques in healthy eating and physical activity interventions: a meta-regression. Health Psychol.

[52] Mann T, de Ridder D, Fujita K (2013). Self-regulation of health behavior: social psychological approaches to goal setting and goal striving. Health Psychol.

[53] Walton A, Nahum-Shani I, Crosby L, Klasnja P, Murphy S (2018). Optimizing digital integrated care via micro-randomized trials. Clin Pharmacol Ther.

[54] Rabin C, Bock B (2011). Desired features of smartphone applications promoting physical activity. Telemed J E Health.

[55] Chang TR, Kaasinen E, Kaipainen K (2012). What influences users' decisions to take apps into use?: a framework for evaluating persuasive and engaging design in mobile Apps for well-being. Proceedings of the 11th International Conference on Mobile and Ubiquitous Multimedia.

[56] Dennison L, Morrison L, Conway G, Yardley L (2013). Opportunities and challenges for smartphone applications in supporting health behavior change: qualitative study. J Med Internet Res.

[57] Ehlers DK, Huberty JL (2014). Middle-aged women's preferred theory-based features in mobile physical activity applications. J Phys Act Health.

[58] Gowin M, Cheney M, Gwin S, Franklin Wann T (2015). Health and fitness app use in college students: a qualitative study. Am J Health Educ.

[59] Middelweerd A, van der Laan DM, van Stralen MM, Mollee JS, Stuij M, te Velde SJ, Brug J (2015). What features do Dutch university students prefer in a smartphone application for promotion of physical activity? A qualitative approach. Int J Behav Nutr Phys Act.

[60] Miyamoto SW, Henderson S, Young HM, Pande A, Han JJ (2016). Tracking health data is not enough: a qualitative exploration of the role of healthcare partnerships and mHealth technology to promote physical activity and to sustain behavior change. JMIR Mhealth Uhealth.

[61] Baretta D, Perski O, Steca P (2019). Exploring users' experiences of the uptake and adoption of physical activity apps: longitudinal qualitative study. JMIR Mhealth Uhealth.

[62] Lyons EJ, Lewis ZH, Mayrsohn BG, Rowland JL (2014). Behavior change techniques implemented in electronic lifestyle activity monitors: a systematic content analysis. J Med Internet Res.

[63] Brannon EE, Cushing CC (2015). A systematic review: is there an app for that? Translational science of pediatric behavior change for physical activity and dietary interventions. J Pediatr Psychol.

[64] Williams SL, French DP (2011). What are the most effective intervention techniques for changing physical activity self-efficacy and physical activity behaviour--and are they the same?. Health Educ Res.

[65] Olander EK, Fletcher H, Williams S, Atkinson L, Turner A, French DP (2013). What are the most effective techniques in changing obese individuals' physical activity self-efficacy and behaviour: a systematic review and meta-analysis. Int J Behav Nutr Phys Act.

[66] O'Brien N, McDonald S, Araújo-Soares V, Lara J, Errington L, Godfrey A, Meyer TD, Rochester L, Mathers JC, White M, Sniehotta FF (2015). The features of interventions associated with long-term effectiveness of physical activity interventions in adults aged 55-70 years: a systematic review and meta-analysis. Health Psychol Rev.

[67] Samdal GB, Eide GE, Barth T, Williams G, Meland E (2017). Effective behaviour change techniques for physical activity and healthy eating in overweight and obese adults; systematic review and meta-regression analyses. Int J Behav Nutr Phys Act.

[68] Michie S, van Stralen MM, West R (2011). The behaviour change wheel: a new method for characterising and designing behaviour change interventions. Implement Sci.

[69] Bort-Roig J, Gilson ND, Puig-Ribera A, Contreras RS, Trost SG (2014). Measuring and influencing physical activity with smartphone technology: a systematic review. Sports Med.

[70] Dute DJ, Bemelmans WJ, Breda J (2016). Using mobile apps to promote a healthy lifestyle among adolescents and students: a review of the theoretical basis and lessons learned. JMIR Mhealth Uhealth.

[71] Abraham C, Michie S (2008). A taxonomy of behavior change techniques used in interventions. Health Psychol.

[72] Michie S, Ashford S, Sniehotta FF, Dombrowski SU, Bishop A, French DP (2011). A refined taxonomy of behaviour change techniques to help people change their physical activity and healthy eating behaviours: the CALO-RE taxonomy. Psychol Health.

[73] Michie S, Wood CE, Johnston M, Abraham C, Francis JJ, Hardeman W (2015). Behaviour change techniques: the development and evaluation of a taxonomic method for reporting and describing behaviour change interventions (a suite of five studies involving consensus methods, randomised controlled trials and analysis of qualitative data). Health Technol Assess.

[74] Carver CS, Scheier MF (1998). On the Self-Regulation of Behavior.

[75] Morton K, Sutton S, Hardeman W, Troughton J, Yates T, Griffin S, Davies M, Khunti K, Eborall H (2015). A text-messaging and pedometer program to promote physical activity in people at high risk of type 2 diabetes: the development of the PROPELS follow-on support program. JMIR Mhealth Uhealth.

[76] Domin A, Ouzzahra Y, Vögele C (2022). Features and components preferred by adolescents in smartphone apps for the promotion of physical activity: focus group study. JMIR Hum Factors.

[77] Kowalski KC, Crocker PR, Donen RM (2004). The Physical Activity Questionnaire for Older Children ( PAQ-C ) and Adolescents ( PAQ-A ) Manual. University of Saskatchewan.

[78] Biddle SJ, Gorely T, Pearson N, Bull FC (2011). An assessment of self-reported physical activity instruments in young people for population surveillance: project ALPHA. Int J Behav Nutr Phys Act.

[79] (2021). What is Fitabase?. Small Steps Labs.

[80] (2021). About RStudio. RStudio.

[81] Chaput JP, Willumsen J, Bull F, Chou R, Ekelund U, Firth J, Jago R, Ortega FB, Katzmarzyk PT (2020). 2020 WHO guidelines on physical activity and sedentary behaviour for children and adolescents aged 5-17 years: summary of the evidence. Int J Behav Nutr Phys Act.

[82] Kramer JN, Künzler F, Mishra V, Smith SN, Kotz D, Scholz U, Fleisch E, Kowatsch T (2020). Which components of a smartphone walking app help users to reach personalized step goals? Results from an optimization trial. Ann Behav Med.

[83] Lubans DR, Smith JJ, Skinner G, Morgan PJ (2014). Development and implementation of a smartphone application to promote physical activity and reduce screen-time in adolescent boys. Front Public Health.

